# Effect of Autoimmune Thyroid Disease on Pregnancy Outcomes: A Systematic Review and Meta-Analysis

**DOI:** 10.3390/jcm14238520

**Published:** 2025-12-01

**Authors:** Anwar A. Sayed, Maryam Mohammed Abdulaal, Elaf Mohammed Emam, Laila Mohammed Daftardar, Razan Essam Kurdi, Yara Basim Alahmadi, Mayes Mohammed Alharbi, Razna Moustafa Aloufi

**Affiliations:** 1 Department of Basic Medical Sciences, College of Medicine, Taibah University, Madinah 42353, Saudi Arabia; 2 Medical Intern, Taibah University, Madinah 42351, Saudi Arabia; 3 College of Medicine, Taibah University, Madinah 42353, Saudi Arabia

**Keywords:** Hashimoto’s thyroiditis, thyroid peroxidase antibodies, subclinical hypothyroidism, preterm delivery, miscarriage, maternal complications

## Abstract

**Background:** Autoimmune thyroid disease (AITD) is common in women of reproductive age and is characterized by thyroid-specific autoantibodies, mainly TPOAbs and TgAbs. Its impact on pregnancy outcomes is not fully understood. However, evidence suggests a potential association with adverse maternal and neonatal outcomes. **Objective**: To assess the association between AITD and adverse pregnancy outcomes and evaluate the effect of levothyroxine (LT4) therapy in high-risk populations. **Methods**: A systematic search of PubMed and Web of Science was performed per PRISMA guidelines. Randomized controlled trials (RCTs) on pregnancy outcomes in women with AITD were included. Primary outcomes were preterm delivery, miscarriage, and live birth; secondary outcomes included maternal and neonatal complications. Risk of bias was assessed using RoB 2.0, and pooled odds ratios (ORs) with 95% confidence intervals (CIs) were calculated. **Results**: Eight RCTs with TPOAb-positive euthyroid or subclinical hypothyroid women were included. AITD increased preterm delivery risk (pooled OR 3.92, 95% CI 2.54–6.05). Miscarriage risk showed high heterogeneity (pooled OR 1.27, 95% CI 0.16–9.82). LT4 reduced miscarriage (pooled OR 0.52, 95% CI 0.34–0.80) and preterm delivery (pooled OR 0.37, 95% CI 0.17–0.80). Live birth rates favored LT4 but were not statistically significant. Maternal and neonatal complications were inconsistently reported. **Conclusions**: AITD is associated with higher preterm delivery risk. LT4 in high-risk women may reduce miscarriage and preterm birth. Further RCTs should stratify by AITD subtype, antibody titer, and thyroid function, and report perinatal outcomes systematically.

## 1. Introduction

Autoimmune thyroid disease (AITD) occurs when the immune system malfunctions, resulting in an attack on the thyroid gland; these disorders are specific to the organ and are primarily mediated by T cells. AITD has a prevalence estimated between 5% and 14% in the general adult population, while the occurrence of antithyroid antibodies may be even more common [1,2]. The two main forms of AITDs are Graves’ disease (GD) and Hashimoto’s thyroiditis (HT), both of which are marked by the infiltration of lymphocytes into the thyroid tissue [3]. AITD is defined by the presence of anti-thyroid antibodies against thyroperoxidase (TPOAbs) and/or antibodies against thyroglobulin (TgAbs), regardless of the thyroid’s functional state, with variations depending on geographical and demographical factors [1,2].

Disturbances in thyroid function have been associated with reproductive issues in women, leading to irregular menstrual cycles, infertility, adverse pregnancy outcomes, and gynecological problems like premature ovarian insufficiency and polycystic ovary syndrome. The intricate molecular interactions between hormones that regulate both thyroid and reproductive functions are further complicated by the connection between certain prevalent autoimmune conditions and disorders affecting the thyroid as well as the hypothalamic–pituitary–gonadal axes [4]. AITDs impact 11% of women of reproductive age [5], and a prevalence of between 2% and 5% in pregnant women [6]. During pregnancy, the maternal thyroid gland faces several metabolic, hemodynamic, and immunologic changes [7]. It has a clinical impact on pregnancy and the postpartum period, along with its impact on fetal health. It is clinically significant in terms of spontaneous abortion, prematurity, gestational diabetes mellitus (GDM), low birth weight [8], or large birth weight and placental weight [9], increased perinatal mortality [10], preterm delivery [11,12], and postpartum thyroiditis [13].

Thyroid hormones are essential during pregnancy for maternal adaptation and fetal growth. In early gestation, the fetus depends entirely on maternal thyroid supply [14]. Even mild disruption can result in miscarriage, preterm birth, and lasting neurodevelopmental problems. These effects may be permanent, highlighting the importance of maintaining thyroid health during pregnancy [15]. Early recognition and management of AITD are crucial for achieving the best outcomes for both the mother and her child. According to the American Thyroid Association guidelines, routine screening for thyroid disease during pregnancy is not recommended; instead, a targeted approach is advised. Instead, they advocate for a targeted screening approach that focuses on women who are at an increased risk of thyroid dysfunction, such as those with elevated TSH (≥2.5 mIU/L), positive thyroid autoantibodies, or a history of thyroid disease or autoimmune conditions [16]. As a result, it is crucial to comprehend how AITD affects pregnancy outcomes. This systematic review and meta-analysis strive to consolidate existing research to elucidate these relationships and offer an in-depth evaluation of the effects of AITD on both maternal and fetal health outcomes.

## 2. Materials and Methods

### 2.1. Study Design

This study was designed and conducted in accordance with the PRISMA (Preferred Reporting Items for Systematic Reviews and Meta-Analyses) guidelines. A systematic review and meta-analysis were performed to evaluate the association between autoimmune thyroid disease (AITD) and pregnancy outcomes. The completed PRISMA checklist is included in the Supplementary Table S1.

### 2.2. Eligibility Criteria

We included studies that evaluated pregnant women diagnosed with AITD, including those TPOAb positive and/or TgAb, with or without subclinical hypothyroidism (SCH). Eligible studies investigated the administration of LT4 therapy administered before or during pregnancy, compared with placebo, no treatment, or euthyroid antibody-negative controls. The studies that reported at least one pregnancy complication outcome, including miscarriage, preterm birth, live birth, pre-eclampsia, gestational diabetes, and placental abruption, as well as neonatal outcomes such as birth weight, NICU admission, and thyroid function assessments, were also included.

Exclusion criteria comprised case reports, editorials, letters, reviews, and animal studies. Non-English publications, duplicate datasets, studies without a control group, studies lacking extractable outcome data relevant to the PICO framework, and studies involving non-pregnant populations or women with overt thyroid dysfunction.

This review considered both studies that compared outcomes in women with AITD versus those without, as well as trials that evaluated the effect of LT4 therapy compared with no treatment or placebo in AITD-related populations.

### 2.3. Information Sources and Search Strategy

A comprehensive literature search was conducted in PubMed and Web of Science databases. The search strategy combined relevant keywords and Medical Subject Headings (MeSH) terms, including: “autoimmune thyroid disease,” “Hashimoto,” “Graves,” “pregnancy outcomes,” “miscarriage,” “preterm birth,” and related terms. Boolean operators (AND, OR) were used to optimize sensitivity and specificity.

### 2.4. Study Selection and Data Extraction

Two authors independently screened the titles and abstracts of the retrieved records, followed by a full-text assessment of potentially eligible studies. Data extraction was also performed independently by two authors using a standardized form. Any disagreements were resolved through discussion, and if a consensus could not be reached, a third author would adjudicate. The standardized data collection sheet, capturing:

First author, year of publication, and study location, study design, and baseline demographic/clinical characteristics, type of AITD studied, reported pregnancy outcomes, Effect estimates (adjusted and unadjusted), such as odds ratios (ORs) and relative risks (RRs).

### 2.5. Quality Assessment

The methodological quality and risk of bias of included RCTs were assessed using the Cochrane Risk of Bias 2.0 (RoB 2.0) tool [17]. The resulting scores guided the interpretation of study rigor, and sensitivity analyses were conducted prioritizing high-quality studies to ensure robustness of findings.

### 2.6. Certainty Assessment

The certainty of evidence for each outcome was assessed using the GRADE tool, with formal certainty ratings (high, moderate, low, very low). The tool provides a structured way to judge risk-of-bias, inconsistency, indirectness, imprecision, and other considerations (including publication/small-study effects).

### 2.7. Data Analysis

Meta-analyses were performed using Review Manager (RevMan) version 5.4 (Cochrane Collaboration, Copenhagen, Denmark). Pooled odds ratios (ORs) with corresponding 95% confidence intervals (CIs) were calculated for each outcome, using a random-effects model [18]. The I^2^ statistic was used to assess statistical heterogeneity in the meta-analysis, with values of <25% considered negligible, 25–75% moderate, and >75% indicating high heterogeneity, where a *p*-value < 0.05 was considered statistically significant heterogeneity. This review summarized outcomes comparing women with AITD versus non-AITD populations (association analyses). Second, we present outcomes from trials evaluating LT4 treatment versus no treatment/placebo in women with AITD, SCH, or TPOAb^+^ (intervention analyses). Currently, the study protocol is not registered and does not have a registration number.

## 3. Results

### 3.1. Study Selection

**Figure 1:**
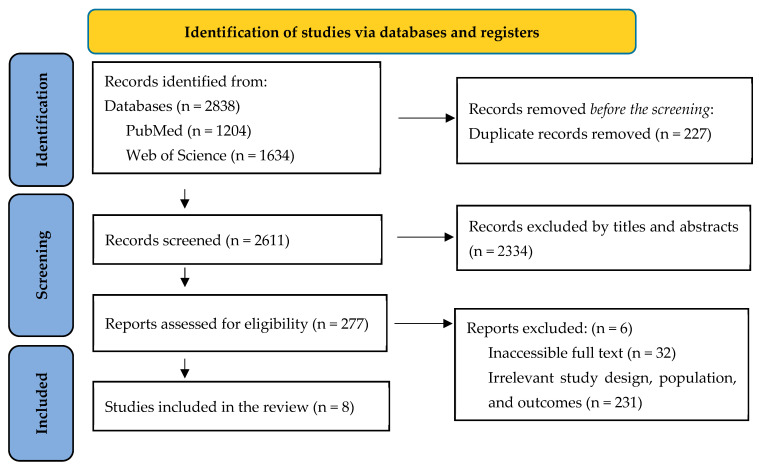
Schematic representation of the criteria for selecting studies in the systematic review.

Studies were extracted from two databases (PubMed and Web of Science). Of these, eight studies were considered suitable for the systematic review (Figure 1).

### 3.2. Study Characteristics

A total of eight studies met the inclusion criteria, comprising RCTs, several of which were double-blind and multicenter. Geographically, studies were conducted across Europe (the Netherlands, Belgium, Denmark, Italy) [19,20,21] and Asia (China, Iran, Pakistan, South Korea) [22,23,24,25,26]. Mean maternal age spanned the mid-20s to mid-30s across studies. Study settings spanned natural conception, recurrent pregnancy loss (RPL) clinics, and assisted reproduction technology (ART) cohorts. Key exclusions were overt thyroid dysfunction, multiple gestations, and major comorbidities (e.g., antiphospholipid syndrome, uncontrolled diabetes). Most trials enrolled women with AITD, defined by either an antibody-positive euthyroid phenotype (TPOAb^+^ with normal TSH), and/or a Subclinical hypothyroidism (SCH) phenotype (raised TSH with normal FT4).

Some studies included mixed populations or ran parallel arms for each category. Anti-TPO titre thresholds varied: ≥9 IU/mL in China, >100 kIU/L in Italy. ART-based studies almost exclusively enrolled euthyroid TPOAb^+^ women, including a study that enrolled euthyroid TPOAb^+^ women with ≥2 losses, while another stratified by both RPL status and thyroid phenotype (SCH vs. euthyroid TPOAb^+^), providing parallel “normal pregnancy” comparators. In intervention studies, LT4 therapy was started pre-conception or early in pregnancy, and continued throughout pregnancy or until pregnancy loss, and has been compared to placebo or no treatment. In ART-specific trials, LT4 was initiated before ovarian stimulation, whereas natural conception groups often began after confirming early pregnancy. This intervention aims to assess how LT4 therapy affects pregnancy outcomes for women with AITD or SCH (Table 1; full version including all studies is provided in Supplementary Table S2).

This review considered the analysis of both studies that compared outcomes in women with AITD versus those without, as well as trials that evaluated the effect of LT4 therapy compared with no treatment or placebo in AITD-related populations.

### 3.3. Preterm Delivery Outcome

Regarding association analyses, across the three RCT cohorts, preterm delivery occurred more frequently in women with AITD than in those without. Rates were 15% versus 6.1% in Leng’s study 24.1% [23], versus 5.6% in Nazarpour’s [25], and 22.4% versus 8.2% in Negro’s study [20], reflecting consistent absolute excesses of roughly 9–18%.

Regarding intervention, when analyses were restricted to AITD-related high-risk phenotypes randomized to LT4 treatment or no treatment, the largest reductions in preterm birth were seen in TPOAb-positive euthyroid women in Nazarpour’s study. (7.1% vs. 23.7%) [25], and in Negro’s [20] (7% vs. 22.4%), as well as in the RPL + SCH subgroup in Leng study (11.9% vs. 35.3%) [23], with other subgroups showing smaller or no differences (Table 2; full version including all studies is provided in Supplementary Table S3).

### 3.4. Pregnancy Loss Outcome

Regarding association, miscarriage findings were inconsistent in women with AITD compared to those without. Leng study [23]. observed lower rates in AITD (7.5% vs. 19.1%), Nazarpour study [25]. found minimal difference (3.4% vs. 4.3%), while Negro study [20]. reported higher miscarriage in the AITD group (13.8% vs. 2.4%).

However, regarding intervention analysis, LT4 across most intervention-eligible RCTs was linked to lower miscarriage rates in high-risk groups such as RPL, SCH, or TPOAb^+^ women, with absolute reductions of about 12–37%. These included 23% vs. 33% in Van Dijk study [19], 60.2% vs. 78.5% in Riaz study [22]., and marked drops in Leng et al.’s RPL + SCH (21.4% vs. 39.7%) and RPL + TPOAb^+^ (7.1% vs. 26.8%) strata; however, in Leng’s study [23] normal pregnancy cohorts, differences were small or favored control (e.g., 21.4% vs. 19.1% in Normal + SCH, 9.7% vs. 5.7% in Normal + TPOAb^+^), and Nazarpour study [25]. showed no difference (3.6% vs. 3.4%). ART-based cohorts by Kim study [26] and Negro study [20]. showed consistent reductions, with Kim study [26]. reporting 0% miscarriage in treated SCH + TPOAb^+^ women versus 33.3% in controls and Negro et al. (3.5% vs. 13.8%) (Table 2; full version including all studies is provided in Supplementary Table S3).

### 3.5. Placental Abruption Outcome

Placental abruption was rare across studies, occurring in none or only isolated cases, with no clear pattern of higher risk in AITD (Table 3; full version including all studies is provided in Supplementary Table S4).

### 3.6. Live Birth Rates and Ongoing Pregnancy

Live birth generally favored LT4, particularly in Riaz study [22], Kim study [26], Negro study [20], and Leng’s study [23] RPL strata, while normal strata showed minimal change. Ongoing pregnancy patterns mirrored live birth trends. (Supplementary Table S3).

### 3.7. Maternal Complications

Based on association analysis, maternal complications, including hypertensive disorders (gestational hypertension (GHTN), pre-eclampsia (PE)), placental abruption, gestational diabetes mellitus (GDM), small-for-gestational-age (SGA) infants, pre-labor rupture of membranes (PROM), and macrosomia, were infrequently reported across the included RCTs (Table 3; full version including all studies is provided in Supplementary Table S4). The event rates were generally low and showed no consistent pattern of increased risk in women with AITD.

Regarding intervention, LT4 therapy did not show consistent reductions in these complications. Leng et al. rates of placental abruption, GHTN, and PE were similar between LT4-treated and control groups across both RPL and normal pregnancy.

### 3.8. Neonatal Outcomes

Neonatal outcomes, such as asphyxia neonatorum, neonatal intensive care unit (NICU) admission, 28-day neonatal survival, birth anthropometrics (weight, head circumference, length), and neonatal thyroid function, were also reported, with no clear pattern of increased risk in both analyses of AITD populations and LT4-related differences. (Table 4; full version including all studies is provided in Supplementary Table S5).

In Leng study [23]., the incidence of asphyxia neonatorum was low and did not differ meaningfully between the LT4 and control groups in either the RPL or normal pregnancy cohorts. Amiri study [24]. and Nazarpour study [25]. provided more detailed neonatal anthropometric and thyroid function data, but these showed no consistent LT4-related differences.

### 3.9. Preterm Delivery Rates: AITD Patients vs. Non-AITD Patients

**Figure 2:**
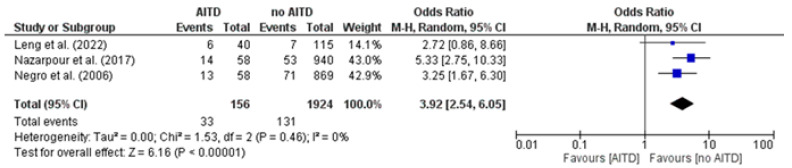
Forest plot illustrates the association between autoimmune thyroid disease (AITD) and preterm delivery, in the included studies [20,23,25].

A pooled meta-analysis was conducted to assess the association between AITD and the risk of preterm delivery across three studies. All studies showed an increased odds ratio in the AITD group, with a pooled OR of 3.92 (95% CI 2.54–6.05), indicating a fourfold higher odds of preterm delivery. Heterogeneity was negligible (I^2^ = 0%), and the test for overall effect was highly significant (Z = 6.16, *p* < 0.00001) (Figure 2).

### 3.10. Placental Abruption: AITD Patients vs. Non-AITD Patients

**Figure 3:**
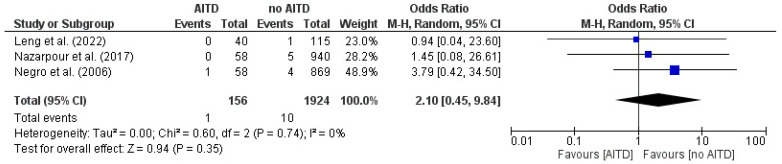
Forest plot illustrating the association between autoimmune thyroid disease (AITD) and placental abruption, in the included studies [20,23,25].

Placental abruption: evaluating the association between AITD and placental abruption in three studies. The pooled OR = 2.10 (95% CI 0.45–9.84) suggests higher odds in the AITD group; however, the estimate was imprecise and not statistically significant, with the CI spanning a decrease to a significant increase. Heterogeneity was negligible (I^2^ = 0%), supporting the consistency of results across studies (Figure 3).

### 3.11. Miscarriage: AITD Patients vs. Non-AITD Patients

**Figure 4:**
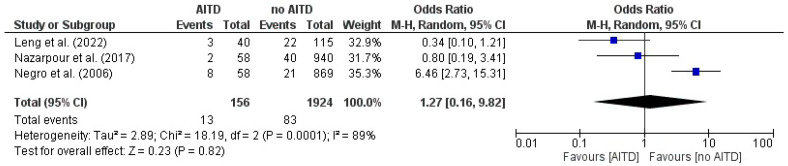
Forest plot illustrates the association between autoimmune thyroid disease (AITD) and the Risk of miscarriage, in the included studies [20,23,25].

However, the Association Between AITD and the Risk of miscarriage in three studies showed mixed results, with Leng study [23]. suggesting a potentially protective effect of AITD (OR 0.34, 95% CI 0.10–1.14), whereas Nazarpour et al. indicated an increased risk (OR 1.88, 95% CI 0.48–7.41), and Negro et al. found a smaller, non-significant increase (OR 1.26, 95% CI 0.96–1.65). The pooled OR = 1.27 (95% CI 0.16–9.82) under a random-effects model reflected high heterogeneity (I^2^ = 89%), indicating that between-study differences dominate over any consistent association. The overall effect was not significant (Z = 0.23, *p* = 0.82) (Figure 4).

### 3.12. Miscarriage: AITD Patients Treated with LT4 vs. Non-Treated Patients

**Figure 5:**
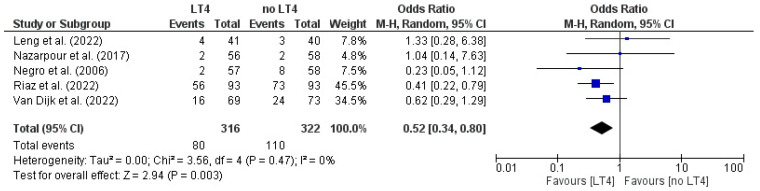
Forest plot illustrating the effect of LT4 therapy versus no treatment on miscarriage, in the included studies [19,20,22,23,25].

The pooled ORs of 0.52 (95% CI 0.34–0.80), corresponding to a 48% relative reduction in odds with LT4, with negligible heterogeneity (I^2^ = 0%) and statistically significant overall effect (Z = 2.94, *p* = 0.003). These findings indicate a consistent and clinically relevant association between LT4 therapy and reduced risk of miscarriage (Figure 5).

### 3.13. Preterm Delivery: AITD Patients Treated with LT4 vs. Non-Treated Patients

**Figure 6:**
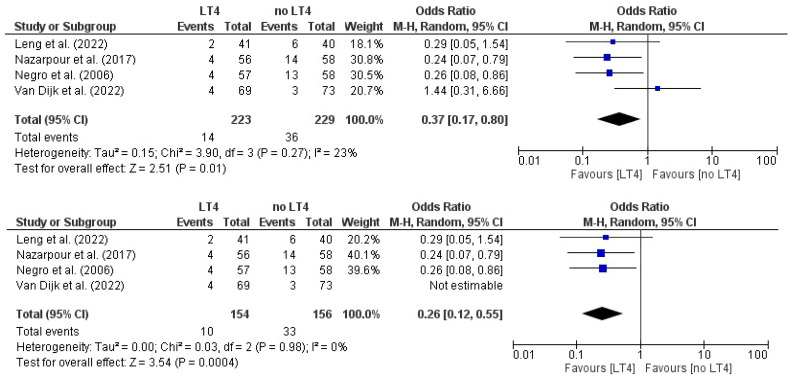
Forest plot illustrating the effect of LT4 therapy versus no treatment on pre-term delivery, in the included studies [19,20,23,25].

The pooled analysis from four RCTs showed an overall OR of 0.37 (95% CI 0.17–0.80), indicating that women receiving LT4 had 63% lower odds of preterm delivery with LT4. The association was statistically significant (Z = 2.51, *p* = 0.01). Heterogeneity was low to moderate (I^2^ = 23%). Sensitivity analysis, restricting to the three studies in the sensitivity set, yielded a pooled OR of 0.26 (95% CI 0.12–0.55), also statistically significant (Z = 3.54, *p* = 0.0004) and with no observed heterogeneity (I^2^ = 0%) (Figure 6).

### 3.14. Live Birth: AITD Patients Treated with LT4 vs. Non-Treated Patients

**Figure 7:**
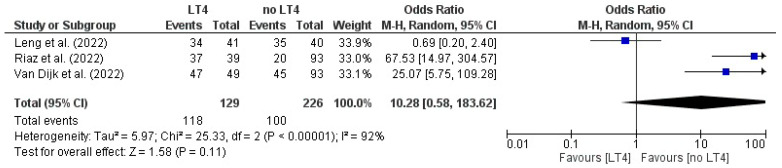
Forest plot illustrating the effect of LT4 therapy versus no treatment on mortality/live birth, in the included studies [19,22,23].

The pooled analysis from three RCTs yielded an overall OR of 10.28 (95% CI 0.58–182.63) for the event with LT4 therapy versus no treatment, equivalent to a tenfold higher odds in the LT4 group. Although point estimates in individual trials favored LT4, the confidence interval around the pooled effect was wide and crossed the line of no effect, and the association was not statistically significant (Z = 1.58, *p* = 0.11). Heterogeneity was considerable (I^2^ = 92%), reflecting large variability in effect sizes across studies (Figure 7).

### 3.15. Risk of Bias Assessment

**Figure 8:**
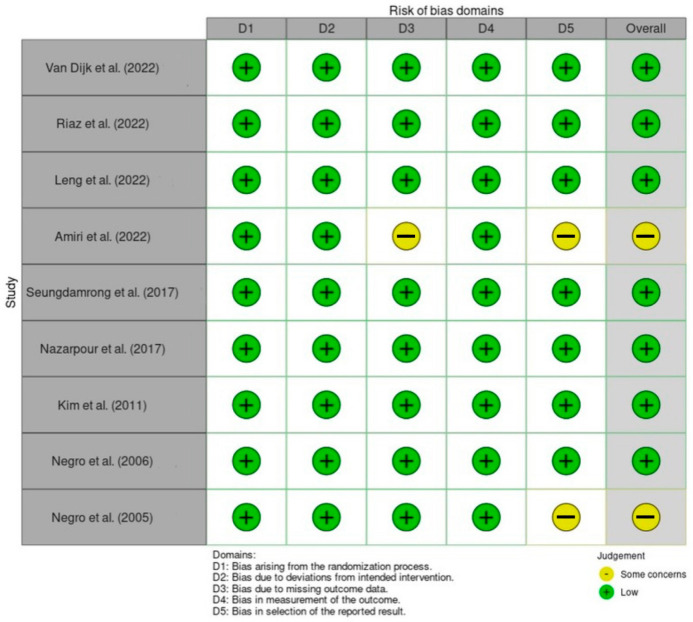
Cochrane Risk of Bias 2.0 (RoB 2.0) quality assessment of the included RCT studies. The figure summarizes the assessment of the main domains of the included studies [18,19,20,21,22,23,24,25,26].

All trials demonstrated a low risk of bias in the domains D1–4, reflecting adequate randomization, adherence to intervention protocols, complete or near-complete outcome data, and objective outcome measurements. For D5, seven trials were rated as low risk, while two studies had “some concerns” due to potential selective reporting. Overall, seven trials were judged as low risk of bias and two as having some concerns, with no high-risk ratings in any domain, supporting the internal validity of the evidence base (Figure 8).

### 3.16. Certainty Assessment

The certainty of evidence for each analysis outcome was assessed using GRADE assessments. The association analyses found moderate-certainty evidence that AITD increases the risk of preterm delivery, while evidence linking AITD to miscarriage and to placental abruption was very low certainty. For intervention analyses, the pooled evidence that LT4 reduces miscarriage and preterm delivery was rated low certainty, and the pooled evidence for an effect of LT4 on live birth was rated very low certainty. Full domain judgments and overall certainty ratings are reported in Supplementary Tables S6 and S7.

## 4. Discussion

In this systematic review and meta-analysis of RCTs and high-quality prospective studies, AITD in women was consistently associated with higher risks of adverse outcomes, particularly preterm delivery and miscarriage in the included RCTs. Women with AITD had an absolute excess risk of preterm delivery ranging from 9% to 18%. This aligns with prior meta-analyses showing a two-fold increase in preterm birth risk among TPOAb-positive women, independent of maternal age and parity, and more than a three-fold increase in miscarriage risk [27]. Additionally, a recent multi-center observational study showed elevated odds for preterm birth, pregnancy-induced hypertension, GDM, and neonatal intensive care admission in antibody-positive women [28]. These findings reinforce the concept that thyroid autoimmunity exerts effects beyond overt hypothyroidism, potentially via immune-mediated placental dysfunction and altered vascular adaptation [29].

Importantly, LT4 supplementation in high-risk AITD phenotypes was associated with substantial reductions in preterm birth. Notably, in TPOAb-positive euthyroid women, both Nazarpour study [25] and Negro study [20]. reported absolute risk reductions exceeding 15% [20,25]. Similarly, in Leng et al.’s subgroup of RPL with SCH, LT4 was associated with a 23% lower incidence of preterm delivery [23]. These findings are consistent with recent studies suggesting that optimizing maternal thyroid reserve, even without overt hypothyroidism, may reduce risks of placental dysfunction and immune dysregulation [30,31]. However, not all subgroups derived significant benefits, indicating that treatment effects may be context-dependent and highlighting the need for phenotype-tailored intervention strategies. It is key to acknowledge that the findings of this meta-analysis is an aggregate of several clinical trials, of which some may not necessarily confirm this study’s results, but rather contradict it [32].

The association between AITD and other adverse maternal outcomes, including gestational hypertension and placental abruption, was less consistent in our synthesis. Few included trials systematically evaluated neonatal outcomes beyond birthweight and Apgar scores, echoing similar data gaps noted in observational syntheses. This paucity of neonatal follow-up data constrains the interpretation of the broader perinatal impact of AITD and its treatment [33].

Methodologically, our review applied rigorous eligibility criteria, excluding secondary analyses, and stratified outcomes by functional status. However, variability in defining high-risk phenotypes (e.g., inconsistent TPOAb cut-offs, non-uniform TgAb reporting) likely contributed to heterogeneity. As recommendations emphasize, standardization of autoimmune thyroid definitions and thresholds is essential to enhance the comparability and reproducibility of future trials [34]. Furthermore, screening for autoimmune thyroid disorders after spontaneous abortion is cost-saving, and it improves the subsequent pregnancy rate, as previously published [35].

Our findings should be interpreted in light of several limitations. First, the available evidence base is limited: few RCTs specifically addressed Graves’ disease, most trials focused on TPOAb^+^ or SCH populations, and many outcomes were reported inconsistently, particularly neonatal endpoints. The small quantity of trials for some outcomes, variable lengths of follow-up, and differences in the risk profiles of populations might compromise the accuracy of the combined estimates. Additionally, the approach to stratifying by antibody titre thresholds was not consistent, and the exploration of the relationship between antibody levels and outcomes in terms of dose response was seldom addressed. Furthermore, many cohorts disproportionately included women conceiving via assisted reproduction, which may limit generalizability to spontaneous conceptions. In addition, our review is limited by reliance on published data, which may be subject to reporting bias, and by the small number of trials available for some outcomes. Nevertheless, strengths include a comprehensive multi-database search, adherence to PRISMA 2020 [36], and duplicate screening and extraction, which enhance the transparency and reproducibility of our findings.

## 5. Conclusions

This systematic review and meta-analysis of RCTs demonstrates that AITD in pregnancy is associated with an increased risk of preterm delivery, with inconsistent evidence regarding miscarriage and other adverse maternal outcomes. LT4 supplementation in high-risk phenotypes, particularly euthyroid women with positive thyroid antibodies and a history of RPL or SCH, may reduce preterm birth rates, although benefits for miscarriage prevention remain uncertain. Current evidence is limited by under-representation of Graves’ disease, inconsistent outcome definitions, and sparse neonatal follow-up data. These findings underscore the need for rigorously designed, adequately powered RCTs that stratify by AITD subtype, antibody titre, and pre-pregnancy thyroid function, and that extend follow-up into the neonatal period. Until such data are available, clinical decision-making should balance the potential benefits of targeted thyroid hormone therapy with the uncertainties highlighted in this review.

## Figures and Tables

**Table 1:** Characteristics of Included Studies Evaluating the Effect of AITD and Its Management on Pregnancy Outcomes.

Author, Year	Country	Randomization	Sample Size	Maternal Age (Mean ± SD)	AITD Type	Thyroid Status
Van Dijk et al. (2022) [19]	Netherlands, Belgium, and Denmark	A: LT4-treated TPOAb^+^ B: Untreated TPOAb^+^	Total: 187 A: 94 B: 93	A: 34.9 ± 4.2 B: 33.7 ± 4.7	TPOAb^+^	Euthyroid
Riaz et al. (2022) [22]	Lahore	A: LT4-treated with SCH B: Untreated with SCH	Total: 186 A: 93 B: 93	A: 27.18 ± 2.68 B: 27.38 ± 2	NR	SCH
Leng et al. (2022) [23]	China	PRL group: RPL + SCH LT4 Control RPL + TPOAb^+^ LT4 Control Normal group: Normal + SCH LT4 Control Normal + TPOAb^+^ LT4 Control	PRL group RPL + SCH: LT4 = 131 Control = 136 RPL + TPOAb^+^: LT4 = 42 Control = 41 Normal group Normal + SCH LT4 = 112 Control = 115 Preterm delivery	PRL group RPL + SCH: LT4 = 29.52 ± 3.75 Control = 29.58 ± 3.51 RPL + TPOAb^+^: LT4 = 28.72 ± 3.74 Control = 29.64 ± 3.98 Normal group Normal + SCH LT4 = 28.62 ± 3.52 Control = 28.53 ± 3.64 Normal + TPOAb^+^ LT4 = 28.64 ± 3.02 Control = 28.40 ± 2.57	TPOAb^+^ and/or SCH	SCH or euthyroid with TPOAb^+^
Amiri et al. (2022) [24]	Iran	A: LT4-treated with SCH and TPOAb^+^ B: Untreated with SCH and TPOAb^+^	Total: 2277	27.70 ± 4.99	TPOAb^+^	Euthyroid and SCH
Nazarpour et al. (2017) [25]	Iran	A: LT4-treated TPOAb^+^ B: Untreated TPOAb^+^ C: Euthyroid TPOAb^−^	Total: 1159 A: 65 B: 66 C: 1028	A: 26.6 ± 5.82 B: 27.0 ± 4.67 C: 27.1 ± 5.17	TPOAb^+^	Euthyroid and SCH
Kim et al. (2011) [26]	South Korea	A: LT4-treated with SCH and TPOAb^+^ B: Untreated with SCH and TPOAb^+^	Total: 64 A: 32 B: 32 TPOAb^+^ A: 26/32 B: 25/32	A: 36.0 ± 2.4 B: 36.1 ± 2.2	TPOAb^+^ (TGAb status also reported)	SCH
Negro et al. (2006) [20]	Italy	A: LT4-treated TPOAb^+^ B: Untreated TPOAb^+^ C: Euthyroid TPOAb^−^	Total: 984 A: 57 B: 58 C: 869	A: 30 ± 5 B: 30 ± 6 C: 28 ± 5	TPOAb^+^	Euthyroid
Negro et al. (2005) [21]	Italy	A: LT4-treated infertile TPOAb^+^ B: Untreated infertile TPOAb^+^ C: Infertile TPOAb^−^	Total: 484 A: 36 B: 36 C: 412	Total: 30.2 ± 4 A: 29.2 ± 4 B: 29.2 ± 4 C: 30.4 ± 5	TPOAb^+^	Euthyroid

LT4: levothyroxine, NR: Not reported, RPL: Recurrent Pregnancy Loss, SCH: subclinical hypothyroid, TPOAb^+^: thyroid peroxidase antibody positive.

**Table 2:** Pregnancy Outcomes in Studies Evaluating AITD and Pregnancy.

Author, Year	Miscarriage N (%)	Stillbirth N (%)	Preterm Birth N (%)	Mortality/Live Birth N (%)	Ongoing Pregnancy N (%)
Van Dijk et al. (2022) [19]	A: 16/69 (23) B: 24/73 (33)	NR	A: 4/69 (6%) B: 3/73 (4.1)	A: 47/94 (50) B: 45/93 (48.4)	A: 47/69 (68.1) B: 46/73 (63)
Riaz et al. (2022) [22]	A: 56 (60.2) B: 73 (78.5)	NR	NR	A: 37 (39.8) B:20 (21.5)	NR
Leng et al. (2022) [23]	PRL group RPL + SCH: LT4 = 28 (21.4) Control = 54 (39.7) RPL + TPOAb^+^: LT4 = 3 (7.1) Control = 11 (26.8) Normal group Normal + SCH LT4 = 24 (21.4) Control = 22 (19.1) Normal + TPOAb^+^ LT4 = 4 (9.7) Control = 3 (5.7)	NR	PRL group RPL + SCH: LT4 = 11 (11.9) Control = 22 (35.3) RPL + TPOAb^+^: LT4 = 3 (7.9) Control = 3 (10.7) Normal group Normal + SCH LT4 = 2 (2.6) Control = 7 (9.9) Normal + TPOAb^+^ LT4 = 2 (5.9) Control = 6 (17.1)	PRL group RPL + SCH: LT4 = 92 (70.2) Control = 64 (47.1) RPL + TPOAb^+^: LT4 = 38 (90.5) Control = 28 (68.3) Normal group Normal + SCH LT4 = 7 (69.6) Control = 71 (61.7) Normal + TPOAb^+^ LT4 = 34 (82.9) Control = 35 (87.5)	PRL group RPL + SCH: LT4 = 11 (8.4) Control = 18 (13.2) RPL + TPOAb^+^: LT4 = 1 (2.4) Control = 2 (4.9) Normal group Normal + SCH LT4 = 10 (8.9) Control = 22 (19.1) Normal + TPOAb^+^ LT4 = 3 (7.3) Control = 2 (5)
Amiri et al. (2022) [24]	75 (3.3)	Stillbirth: 4 (0.22)	118 (6.56)	NR	NR
Nazarpour et al. (2017) [25]	A: 2 (3.6) B: 2 (3.4) C: 40 (4.3)	A: 0 B: 0 C: 2 (0.2)	A: 4 (7.1) B: 14 (23.7) C: 53 (5.6)	NR	NR
Kim et al. (2011) [26]	A: 0/17 B: 4/12 (33.3)	NR	A: 0/17 B: 1/12	A: 17/32 (53.1) B: 8/32 (25)	NR
Negro et al. (2006) [20]	A: 2 (3.5) B: 8 (13.8) C: 21 (2.4)	NR	A: 4 (7) B: 13 (22.4) C: 71 (8.2)	NR	NR
Negro et al. (2005) [21]	A: 8/24 (33) B: 11/21 (52) C: 82/318 (26)	NR	NR	A: 16/24 B: 10/21 (28) C: 236/318	NR

NR: Not reported, RPL: Recurrent Pregnancy Loss, SCH: subclinical hypothyroid, TPOAb^+^: thyroid peroxidase antibody positive.

**Table 3:** Maternal Complications in Studies Evaluating AITD and Pregnancy.

Author, Year	Placental Abruption N (%)	GHTN N (%)	PE N (%)	GDM N (%)	SGA N (%)	PROM N (%)	Macrosomia N (%)
Leng et al. (2022) [23]	PRL group RPL + SCH: LT4 = 1 (0.7) Control = 1 (0.7) RPL + TPOAb^+^: LT4 = 0 Control = 0 Normal group Normal + SCH LT4 = 0 Control = 1 (0.9) Normal + TPOAb^+^ LT4 = 0 Control = 0	PRL group RPL + SCH: LT4 = 6 (4.6) Control = 3 (2.2) RPL + TPOAb^+^: LT4 = 0 Control = 2 (4.8) Normal group Normal + SCH LT4 = 5 (4.5) Control = 3 (2.7) Normal + TPOAb^+^ LT4 = 2 (4.9) Control = 4 (10)	PRL group RPL + SCH: LT4 = 0 Control = 0 RPL + TPOAb^+^: LT4 = 0 Control = 1 (2.4) Normal group Normal + SCH LT4 = 1 (0.9) Control = 2 (1.7) Normal + TPOAb^+^ LT4 = 0 Control = 0	PRL group RPL + SCH: LT4 = 8 (6.1) Control = 1 (0.7) RPL + TPOAb^+^: LT4 = 4 (9.5) Control = 1 (2.4) Normal group Normal + SCH LT4 = 4 (3.6) Control = 7 (6.1) Normal + TPOAb^+^ LT4 = 2 (4.9) Control = 3 (7.5)	PRL group RPL + SCH: LT4 = 8 (8.7) Control = 3 (4.7) RPL + TPOAb^+^: LT4 = 3 (7.8) Control = 0 Normal group Normal + SCH LT4 = 1 (1.3) Control = 2 (2.8) Normal + TPOAb^+^ LT4 = 2 (5.9) Control = 2 (5.7)	PRL group RPL + SCH: LT4 = 0 Control = 0 RPL + TPOAb^+^: LT4 = 1 (2.4) Control = 0 Normal group Normal + SCH LT4 = 6 (5.4) Control = 1 (0.9) Normal + TPOAb^+^ LT4 = 0 Control = 2 (5)	PRL group RPL + SCH: LT4 = 0 Control = 3 (4.7) RPL + TPOAb^+^: LT4 = 0 Control = 1 (3.6) Normal group Normal + SCH LT4 = 2 (2.6) Control = 7 (8.9) Normal + TPOAb^+^ LT4 = 3 (8.8) Control = 1 (2.9)
Amiri et al. (2022) [24]	15 (0.83)	NR	NR	NR	NR	NR	NR
Nazarpour et al. (2017) [25]	A: 0 B: 0 C: 5 (0.5)	NR	NR	NR	NR	NR	NR
Negro et al. (2006) [20]	A: 0 B: 1 (1.7) C: 4 (0.5)	A: 5 (8.8) B: 7 (12) C: 63 (7.2)	A: 2 (3.5) B: 3 (5.2) C: 32 (3.7)	NR	NR	NR	NR

NR: Not reported, GDM: gestational diabetes mellitus, GHTN: gestational hypertension, PE: pre-eclampsia; PROM: pre-labor rupture of membranes, SGA = small-for-gestational-age, RPL: Recurrent Pregnancy Loss, SCH: subclinical hypothyroid, TPOAb^+^: thyroid peroxidase antibody positive.

**Table 4:** Neonatal Outcomes in Studies Evaluating AITD and Pregnancy.

Author, Year	Neonatal Admission N (%)	Gestational Age Mean (SD)	Asphyxia Neonatorum Mean (SD)	Survival 28 Days of Neonatal Life N (%)	Neonatal Thyroid Function Median (IQR)
Van Dijk et al. (2022) [19]	NR	NR	NR	A: 47/69 (68.1) B: 45/73 (61.6)	NR
Leng et al. (2022) [23]	NR	NR	PRL group RPL + SCH: LT4 = 0 Control = 2 (3.1) RPL + TPOAb^+^: LT4 = 0 Control = 0 Normal group Normal + SCH LT4 = 0 Control = 1 (1.4) Normal + TPOAb^+^ LT4 = 0 Control = 1 (2.9)	NR	NR
Amiri et al. (2022) [24]	147 (8.18)	GA at first week: 11.64 (4.18) GA at delivery: 39.01 (1.67)	NR	NR	Neonate FT41 1st trimester: 2.9 (2.5–3.5) 2nd trimester: 3.3 (2.8–4.0) 3rd trimester: 2.8 (2.4–3.3)
Nazarpour et al. (2017) [25]	A: 2 (3.6) B: 12 (20.7) C: 75 (8.0)	A: 39.3 (1.3) B: 38.4 (1.7) C: 39.4 (1.4)	NR	NR	Neonatal TSH A: 1.3 (0.45–1.9) B: 1.0 (0.43–1.9) C: 0.90 (0.40–1.7)

## Data Availability

Data supporting the findings of this study are available in the included publications referenced in the manuscript. No new datasets were generated. Currently, the study protocol is not registered and does not have a registration number.

## Supplementary Materials

The following supporting information can be downloaded at: https://www.mdpi.com/article/10.3390/jcm14238520/s1, Table S1: PRISMA Checklist 2020; Table S2: Characteristics of Included Studies Evaluating the Effect of AITD and Its Management on Pregnancy Outcomes; Table S3: Pregnancy Outcomes in Studies Evaluating AITD and Pregnancy; Table S4: Maternal Complications in Studies Evaluating AITD and Pregnancy; Table S5: Neonatal Outcomes in Studies Evaluating AITD and Pregnancy; Table S6: Question: [AITD] compared to [No AITD] for [Pregnant women]; Table S7: Question: [AITD Patients treated with LT4] compared to [Non-treated Patients] for [Pregnant women].

## Abbreviations

The following abbreviations are used in this manuscript:

AITD	Autoimmune Thyroid Disease
TPOAb	Thyroid Peroxidase Antibody
TgAb	Thyroglobulin Antibody
LT4	Levothyroxine
RCT	Randomized Controlled Trial
OR	Odds Ratio
IVF	In Vitro Fertilization
NICU	Neonatal Intensive Care Unit
CI	Confidence Interval
