# Supplementary material for: Effect of Autoimmune Thyroid Disease on Pregnancy Outcomes: A Systematic Review and Meta-Analysis
Source: J Clin Med. 2025 Dec 1;14(23):8520. doi: 10.3390/jcm14238520 (PMC12693014; doi:10.3390/jcm14238520)
Supplement: Supplementary file 1 [file jcm-14-08520-s001.zip › jcm-3906648-supplementary.pdf]

Supplementary Table S1: PRISMA Checklist 2020.

Section and Topic	Item #	Checklist item	Location where item is reported
**TITLE**			
Title	1	Identify the report as a systematic review.	1
**ABSTRACT**			
Abstract	2	See the PRISMA 2020 for Abstracts checklist.	1
**INTRODUCTION**			
Rationale	3	Describe the rationale for the review in the context of existing knowledge.	1-2
Objectives	4	Provide an explicit statement of the objective(s) or question(s) the review addresses.	1
**METHODS**			
Eligibility criteria	5	Specify the inclusion and exclusion criteria for the review and how studies were grouped for the syntheses.	2
Information sources	6	Specify all databases, registers, websites, organisations, reference lists and other sources searched or consulted to identify studies. Specify the date when each source was last searched or consulted.	3
Search strategy	7	Present the full search strategies for all databases, registers and websites, including any filters and limits used.	3
Selection process	8	Specify the methods used to decide whether a study met the inclusion criteria of the review, including how many reviewers screened each record and each report retrieved, whether they worked independently, and if applicable, details of automation tools used in the process.	3
Data collection process	9	Specify the methods used to collect data from reports, including how many reviewers collected data from each report, whether they worked independently, any processes for obtaining or confirming data from study investigators, and if applicable, details of automation tools used in the process.	3
Data items	10a	List and define all outcomes for which data were sought. Specify whether all results that were compatible with each outcome domain in each study were sought (e.g. for all measures, time points, analyses), and if not, the methods used to decide which results to collect.	3
	10b	List and define all other variables for which data were sought (e.g. participant and intervention characteristics, funding sources). Describe any assumptions made about any missing or unclear information.	3
Study risk of bias assessment	11	Specify the methods used to assess risk of bias in the included studies, including details of the tool(s) used, how many reviewers assessed each study and whether they worked independently, and if applicable, details of automation tools used in the process.	3
Effect measures	12	Specify for each outcome the effect measure(s) (e.g. risk ratio, mean difference) used in the synthesis or presentation of results.	3
Synthesis methods	13a	Describe the processes used to decide which studies were eligible for each synthesis (e.g. tabulating the study intervention characteristics and comparing against the planned groups for each synthesis (item #5)).	3
	13b	Describe any methods required to prepare the data for presentation or synthesis, such as handling of missing summary statistics, or data conversions.	3
	13c	Describe any methods used to tabulate or visually display results of individual studies and syntheses.	3
	13d	Describe any methods used to synthesize results and provide a rationale for	3

Section and Topic	Item #	Checklist item	Location where item is reported
		the choice(s). If meta-analysis was performed, describe the model(s), method(s) to identify the presence and extent of statistical heterogeneity, and software package(s) used.	
	13e	Describe any methods used to explore possible causes of heterogeneity among study results (e.g. subgroup analysis, meta-regression).	3
	13f	Describe any sensitivity analyses conducted to assess robustness of the synthesized results.	3
Reporting bias assessment	14	Describe any methods used to assess risk of bias due to missing results in a synthesis (arising from reporting biases).	Not applicable
Certainty assessment	15	Describe any methods used to assess certainty (or confidence) in the body of evidence for an outcome.	3
**RESULTS**			
Study selection	16a	Describe the results of the search and selection process, from the number of records identified in the search to the number of studies included in the review, ideally using a flow diagram.	3-4
	16b	Cite studies that might appear to meet the inclusion criteria, but which were excluded, and explain why they were excluded.	3-4
Study characteristics	17	Cite each included study and present its characteristics.	5-8
Risk of bias in studies	18	Present assessments of risk of bias for each included study.	16
Results of individual studies	19	For all outcomes, present, for each study: (a) summary statistics for each group (where appropriate) and (b) an effect estimate and its precision (e.g. confidence/credible interval), ideally using structured tables or plots.	2-4
Results of syntheses	20a	For each synthesis, briefly summarise the characteristics and risk of bias among contributing studies.	5-8,16
	20b	Present results of all statistical syntheses conducted. If meta-analysis was done, present for each the summary estimate and its precision (e.g. confidence/credible interval) and measures of statistical heterogeneity. If comparing groups, describe the direction of the effect.	13-15
	20c	Present results of all investigations of possible causes of heterogeneity among study results.	11,13-14
	20d	Present results of all sensitivity analyses conducted to assess the robustness of the synthesized results.	12, 14-15
Reporting biases	21	Present assessments of risk of bias due to missing results (arising from reporting biases) for each synthesis assessed.	15
Certainty of evidence	22	Present assessments of certainty (or confidence) in the body of evidence for each outcome assessed.	
**DISCUSSION**			
Discussion	23a	Provide a general interpretation of the results in the context of other evidence.	16-17
	23b	Discuss any limitations of the evidence included in the review.	16-17
	23c	Discuss any limitations of the review processes used.	16-17
	23d	Discuss implications of the results for practice, policy, and future research.	16-17
**OTHER INFORMATION**			
Registration and protocol	24a	Provide registration information for the review, including register name and registration number, or state that the review was not registered.	

Section and Topic	Item #	Checklist item	Location where item is reported
	24b	Indicate where the review protocol can be accessed, or state that a protocol was not prepared.	
	24c	Describe and explain any amendments to information provided at registration or in the protocol.	
Support	25	Describe sources of financial or non-financial support for the review, and the role of the funders or sponsors in the review.	17-18
Competing interests	26	Declare any competing interests of review authors.	17-18
Availability of data, code and other materials	27	Report which of the following are publicly available and where they can be found: template data collection forms; data extracted from included studies; data used for all analyses; analytic code; any other materials used in the review.	17

Supplementary Table S2: Characteristics of Included Studies Evaluating the Effect of AITD and Its Management on Pregnancy Outcomes

Author, year	Country	Study design	Randomization	Sample size	Maternal age (Mean ± SD)	Inclusion criteria	AITD type	Thyroid status	Pregnancy type	Mode of delivery N (%)	Intervention
Van Dijk et al. (2022)	Netherlands, Belgium, and Denmark	Double-blind, placebo-controlled, RCT, Multicentre	A: LT4-treated TPOAb⁺ B: Untreated TPOAb⁺	Total: 187<br>A: 94 B: 93	A: 34.9 ± 4.2 B: 33.7 ± 4.7	18–42 y ≥2 pregnancy losses (<20 wks) TPOAb⁺ (>lab ref. range) TSH within institutional reference Natural conception or ART No APS, other autoimmune disease, thyroid disease, previous T4LIFE enrolment, or LT4	TPO Ab+	Euthyroid	Singleton or Assisted ART conception	NR	Oral LT4, Dose: 0.5–1.0 μg/kg by baseline TSH (<1.0 → 0.5 μg/kg; 1.0–2.5 → 0.75 μg/kg; >2.5 → 1.0 μg/kg), rounded to 12.5 μg Timing: pre-conception Duration: through pregnancy

						contraindications					
Riaz et al. (2022)	Lahore	Single-blind, placebo-controlled RCT.	A: LT4-treated with SCH<br>B: Untreated with SCH	Total: 186<br><br>A: 93<br>B: 93	A: 27.18 ± 2.68<br>B: 27.38 ± 2	Age 20–30 years<br>Singleton pregnancy<br>≥3 first-trimester miscarriages<br>SCH<br>No prior thyroid surgery, known thyroid disease on treatment, twin pregnancy, non-thyroid miscarriage causes (e.g., congenital malformations, diabetes, lupus anticoagulant, antiphospholipid antibodies, uterine anomalies)	NR	SCH	Singleton	NR	Dose:<br>Hypothyroidism: LT4 0.5 mg/kg orally daily<br>Hyperthyroidism: Propylthiouracil 0.3 mg/kg orally daily.<br>Timing: Initiated early in pregnancy, biweekly TFT monitoring, dose adjusted to maintain TSH 0.45–4.5 IU/L & fT4 14–20 μmol/L<br>Duration: continued until viability or pregnancy loss <24 wks
Leng et al. (2022)	China	Single-blind RCT, parallel, multicentre	PRL group:<br>RPL+SCH<br>LT4<br>Control<br>RPL+TPOAb+<br>LT4<br>Control	Total: 1736<br><br>PRL group = 861<br>RPL+SCH = 267<br>LT4 = 131	PRL group = 29.12 ± 3.73<br>RPL+SCH:<br>LT4 = 29.52 ± 3.75<br>Control =	Pregnant <12 wks<br>≥2 RPL (groups only)<br>SCH = TSH above pregnancy-specific reference, <10.0 μIU/	TPOAb+ and/or SCH	SCH or euthyroid with TPOAb+	Singleton or Assisted ART conception	VD:<br>PRL group = 280 (45.9)<br>RPL+SCH:<br>LT4 = 38 (41.3)	LT4 50 μg/day orally, started 4–8 days post-first prenatal visit, continued until delivery/miscarriage

			Euthyroid non-randomized<br><br>Normal group:<br>Normal pregnancy + SCH<br>LT4<br>Control<br>Normal pregnancy + TPOAb+<br>LT4<br>Control<br>Euthyroid non-randomized	Control = 136<br>RPL+TPOAb+ = 83<br>LT4 = 42<br>Control = 41<br><br>Normal group = 875<br>Normal pregnancy + SCH =227<br>LT4 = 112<br>Control = 115<br>Normal pregnancy + TPOAb+ = 81<br>LT4 = 41<br>Control = 40	29.58 ± 3.51<br>RPL+TPOAb+:<br>LT4 = 28.72 ± 3.74<br>Control = 29.64 ± 3.98<br><br>Normal group = 28.78 ± 3.24<br>Normal +SCH<br>LT4 = 28.62 ± 3.52<br>Control = 28.53 ± 3.64<br>Normal +TPOAb+<br>LT4 = 28.64 ± 3.02<br>Control = 28.40 ± 2.57	mL, FT4 normal; TPOAb+ ≥ 9 IU/mL, normal TSH;<br>Natural conception<br>Age 18–39<br>Excluded ART, overt thyroid disease, uterine abnormalities, and genetic causes				Control = 39 (60.9)<br>RPL+TPOAb+:<br>LT4 = 22 (57.9)<br>Control = 19 (67.9)<br>Normal group = 454 (65.2)<br>Normal +SCH<br>LT4 = 48 (61.5)<br>Control = 46 (64.8)<br>Normal +TPOAb+<br>LT4 = 24 (70.7)<br>Control = 25 (71.4)<br><br>CS:<br>PRL group = 330 (54.1)<br>RPL+SCH:<br>LT4 = 25 (58.7)<br>Control = 25 (39.1)<br>RPL+TPOAb+:<br>LT4 = 16 (42.1)<br>Control = 9 (32.1)	

										Normal group = 242 (34.8) Normal +SCH LT4 = 30 (38.5) Control = 25 (35.2) Normal +TPOAb+ LT4 = 24 (70.7) Control = 25 (71.4)	
Amiri et al. (2022)	Iran	Single-blind RCT	A: LT4-treated with SCH and TPOAb⁺ B: Untreated with SCH and TPOAb⁺	Total: 2277	27.70 ± 4.99	Pregnancy in the 1st trimester <18 y excluded No overt thyroid disease SCH (TSH 4–10 mIU/L, FTI 1–4.5) and/or euthyroid TPOAb+	TPOAb+	Euthyroid and SCH	Singleton	NR	LT4 oral Dose: 0.5 μg/kg/day if TSH < 1.0; 0.75 μg/kg/day if TSH 1.0–2.0; 1 μg/kg/day if TSH > 2.0 or TPOAb > 1500 IU/mL Timing: 4–8 days after 1st prenatal visit Duration: Continued until delivery
Nazarpour et al. (2017)	Iran	Single-blind RCT, parallel	A: LT4-treated TPOAb⁺ B: Untreated TPOAb⁺ C: Euthyroid TPOAb⁻	Total: 1159 A: 65 B: 66 C: 1028	A: 26.6 ± 5.82 B: 27.0 ± 4.67 C: 27.1 ± 5.17	Pregnant ≤20 wks TPOAb⁺ Normal FT4I No overt hypo/hyperthyroidism Singleton	TPOAb+	Euthyroid and SCH	Singleton	NR	LT4 oral Dose: 0.5–1 μg/kg/d based on baseline TSH or TPOAb > 1500 IU/ml Timing: Started within 4–8 days of enrolment Duration: Continued until delivery

Kim et al. (2011)	South Korea	RCT	A: LT4-treated with SCH and TPOAb⁺<br>B: Untreated with SCH and TPOAb⁺	Total: 64<br><br>A: 32<br>B: 32<br><br>TPOAb+<br>A: 26/32<br>B: 25/32	A: 36.0 ± 2.4<br>B: 36.1 ± 2.2	Infertile women (27–41 y) with SCH TSH > 4.5 mIU/L Normal FT4 No overt hypothyroidism Regular ovulatory cycles Normal cardiac/hepatic/renal function No fertility meds in the prior three months	TPOAb+ (TGAb status also reported)	SCH	Assisted reproduction (IVF/ICSI)	NR	LT4 oral<br>Dose: 50 µg/day<br>Timing: Starting on the first day of COS until the β-hCG test; increased if pregnant to keep TSH < 2.5<br>Duration: continued through pregnancy
Negro et al. (2006)	Italy	RCT	A: LT4-treated TPOAb⁺<br>B: Untreated TPOAb⁺<br>C: Euthyroid TPOAb⁻	Total: 984<br><br>A: 57<br>B: 58<br>C: 869	A: 30 ± 5<br>B: 30 ± 6<br>C: 28 ± 5	Euthyroid at baseline Singleton pregnancy No overt hypo/hyperthyroidism TPOAb+ >100 kIU/L	TPOAb+	Euthyroid	Singleton	NR	LT4 oral<br>Dose: based on TSH,<br>0.5 µg/kg/day if TSH <1.0;<br>0.75 µg/kg/day if TSH 1.0–2.0;<br>1.0 µg/kg/day if TSH >2.0 mIU/L or TPOAb >1500 kIU/L<br>Timing: Started 3–7 days after first OB visit;<br>Duration: Continued until delivery
Negro et al. (2005)	Italy	RCT, parallel	A: LT4-treated infertile TPOAb⁺<br>B: Untreated	Total: 484<br><br>A: 36<br>B: 36<br>C: 412	Total: 30.2 ± 4<br><br>A: 29.2 ± 4<br>B: 29.2 ± 4<br>C: 30.4 ± 5	Infertile, euthyroid women Undergoing the first ART cycle Screened for TPOAb	TPOAb+	Euthyroid	Assisted reproduction (IVF/ICSI)	NR	LT4 oral<br>Dose: 1 µg/kg/day<br>Timing: Starting 1-month pre-ART<br>Duration: continued

			infertile TPOAb$^+$<br>C: Infertile TPOAb$^-$			No overt thyroid dysfunction					through pregnancy

COS: Controlled ovarian stimulation, CS: cesarean delivery, LT4: levothyroxine, NR: Not reported, RPL: Recurrent Pregnancy Loss, SCH: subclinical hypothyroid, TPOAb$^+$: thyroid peroxidase antibody positive, VD: vaginal delivery.

Supplementary Table S3: Pregnancy Outcomes in Studies Evaluating AITD and Pregnancy

Author, year	Miscarriage N (%)	Stillbirth N (%)	Preterm birth N (%)	Mortality/live birth N (%)	Ongoing pregnancy N (%)	Ectopic pregnancy N (%)	Pregnancy of unknown location N (%)	Clinical pregnancy N (%)
Van Dijk et al. (2022)	A: 16/69 (23)<br>B: 24/73 (33)	NR	A: 4/69 (6%)<br>B: 3/73 (4.1)	A: 47/94 (50) B: 45/93 (48.4)	A: 47/69 (68.1)<br>B: 46/73 (63)	NR	A: 4/69 (6)<br>B: 1/73 (1)	NR
Riaz et al. (2022)	A: 56 (60.2)<br>B: 73 (78.5)	NR	NR	A: 37 (39.8)<br>B:20 (21.5)	NR	NR	NR	NR
Leng et al. (2022)	PRL group = 182 (21.1)<br>RPL+SCH:<br>LT4 = 28 (21.4)<br>Control = 54 (39.7)<br>RPL+TPOAb+:<br>LT4 = 3 (7.1)<br>Control = 11 (26.8)<br>Normal group = 81 (9.3)<br>Normal+SCH<br>LT4 = 24 (21.4)<br>Control = 22 (19.1)	NR	PRL group = 85 (13.9)<br>RPL+SCH:<br>LT4 = 11 (11.9)<br>Control = 22 (35.3)<br>RPL+TPOAb+:<br>LT4 = 3 (7.9)<br>Control = 3 (10.7)<br>Normal group = 38 (5.5)<br>Normal+SCH<br>LT4 = 2 (2.6)<br>Control = 7 (9.9)	PRL group = 610 (70.8)<br>RPL+SCH:<br>LT4 = 92 (70.2)<br>Control = 64 (47.1)<br>RPL+TPOAb+:<br>LT4 = 38 (90.5)<br>Control = 28 (68.3)<br>Normal group = 696 (79.5)<br>Normal+SCH<br>LT4 = 7 (69.6)	PRL group = 69 (8)<br>RPL+SCH:<br>LT4 = 11 (8.4)<br>Control = 18 (13.2)<br>RPL+TPOAb+:<br>LT4 = 1 (2.4)<br>Control = 2 (4.9)<br>Normal group = 98 (11.2)<br>Normal+SCH<br>LT4 = 10 (8.9)	NR	NR	NR

Author, year	Miscarriage N (%)	Stillbirth N (%)	Preterm birth N (%)	Mortality/live birth N (%)	Ongoing pregnancy N (%)	Ectopic pregnancy N (%)	Pregnancy of unknown location N (%)	Clinical pregnancy N (%)
	Normal+TPOAb+ LT4 = 4 (9.7) Control = 3 (5.7)		Normal+TPOAb+ LT4 = 2 (5.9) Control = 6 (17.1)	Control = 71 (61.7) Normal+TPOAb+ LT4 = 34 (82.9) Control = 35 (87.5)	Control = 22 (19.1) Normal+TPOAb+ LT4 = 3 (7.3) Control = 2 (5)			
Amiri et al. (2022)	75 (3.3)	Stillbirth: 4 (0.22)	118 (6.56)	NR	NR	NR	NR	NR
Nazarpour (2017)	A: 2 (3.6) B: 2 (3.4) C: 40 (4.3)	A: 0 B: 0 C: 2 (0.2)	A: 4 (7.1) B: 14 (23.7) C: 53 (5.6)	NR	NR	NR	NR	NR
Kim et al. (2011)	A: 0/17 B: 4/12 (33.3)	NR	A: 0/17 B: 1/12	A: 17/32 (53.1) B: 8/32 (25)	NR	NR	NR	A:53.1 (17/32) B: 37.5 (12/32)
Negro et al. (2006)	A: 2 (3.5) B: 8 (13.8) C: 21 (2.4)	NR	A: 4 (7) B: 13 (22.4) C: 71 (8.2)	NR	NR	NR	NR	NR
Negro et al. (2005)	A: 8/24 (33) B: 11/21 (52) C: 82/318 (26)	NR	NR	A: 16/24 B: 10/21 (28) C: 236/318	NR	NR	NR	NR

NR: Not reported, RPL: Recurrent Pregnancy Loss, SCH: subclinical hypothyroid, TPOAb+: thyroid peroxidase antibody positive

Supplementary Table S4: Maternal Complications in Studies Evaluating AITD and Pregnancy

Author, year	Placental Abruption N (%)	GHTN N (%)	PE N (%)	GDM N (%)	SGA N (%)	PROM N (%)	Macrosomia
Van Dijk et al. (2022)	NR	NR	NR	NR	NR	NR	NR

Author, year	Placental Abruption N (%)	GHTN N (%)	PE N (%)	GDM N (%)	SGA N (%)	PROM N (%)	Macrosomia
Riaz et al. (2022)	NR	NR	NR	NR	NR	NR	NR
Leng et al. (2022)	PRL group = 12 (1.4)<br>RPL+SCH:<br>LT4 = 1 (0.7)<br>Control = 1 (0.7)<br>RPL+TPO Ab+:<br>LT4 = 0<br>Control = 0<br><br>Normal group = 9 (1)<br>Normal+SCH<br>LT4 = 0<br>Control = 1 (0.9)<br>Normal+TPOAb+<br>LT4 = 0<br>Control = 0	PRL group = 39 (4.6)<br>RPL+SCH:<br>LT4 = 6 (4.6)<br>Control = 3 (2.2)<br>RPL+TPO Ab+:<br>LT4 = 0<br>Control = 2 (4.8)<br><br>Normal group = 12 (2.4)<br>Normal+SCH<br>LT4 = 5 (4.5)<br>Control = 3 (2.7)<br>Normal+TPOAb+<br>LT4 = 2 (4.9)<br>Control = 4 (10)	PRL group = 18 (2.1)<br>RPL+SCH:<br>LT4 = 0<br>Control = 0<br>RPL+TPO Ab+:<br>LT4 = 0<br>Control = 1 (2.4)<br><br>Normal group = 15 (1.7)<br>Normal+SCH<br>LT4 = 1 (0.9)<br>Control = 2 (1.7)<br>Normal+TPOAb+<br>LT4 = 0<br>Control = 0	PRL group = 76 (8.9)<br>RPL+SCH:<br>LT4 = 8 (6.1)<br>Control = 1 (0.7)<br>RPL+TPO Ab+:<br>LT4 = 4 (9.5)<br>Control = 1 (2.4)<br><br>Normal group = 73 (8.3)<br>Normal+SCH<br>LT4 = 4 (3.6)<br>Control = 7 (6.1)<br>Normal+TPOAb+<br>LT4 = 2 (4.9)<br>Control = 3 (7.5)	PRL group = 22 (3.6)<br>RPL+SCH:<br>LT4 = 8 (8.7)<br>Control = 3 (4.7)<br>RPL+TPO Ab+:<br>LT4 = 3 (7.8)<br>Control = 0<br><br>Normal group = 19 (2.7)<br>Normal+SCH<br>LT4 = 1 (1.3)<br>Control = 2 (2.8)<br>Normal+TPOAb+<br>LT4 = 2 (5.9)<br>Control = 2 (5.7)	PRL group = 39 (4.6)<br>RPL+SCH:<br>LT4 = 0<br>Control = 0<br>RPL+TPO Ab+:<br>LT4 = 1 (2.4)<br>Control = 0<br><br>Normal group = 40 (4.6)<br>Normal+SCH<br>LT4 = 6 (5.4)<br>Control = 1 (0.9)<br>Normal+TPOAb+<br>LT4 = 0<br>Control = 2 (5)	PRL group = 22 (3.6)<br>RPL+SCH:<br>LT4 = 0<br>Control = 3 (4.7)<br>RPL+TPO Ab+:<br>LT4 = 0<br>Control = 1 (3.6)<br><br>Normal group = 45 (6.5)<br>Normal+SCH<br>LT4 = 2 (2.6)<br>Control = 7 (8.9)<br>Normal+TPOAb+<br>LT4 = 3 (8.8)<br>Control = 1 (2.9)
Amiri et al. (2022)	15 (0.83)	NR	NR	NR	NR	NR	NR
Nazarpour (2017)	A: 0<br>B: 0<br>C: 5 (0.5)	NR	NR	NR	NR	NR	NR
Kim et al. (2011)	NR	NR	NR	NR	NR	NR	NR
Negro et al. (2006)	A: 0<br>B: 1 (1.7)<br>C: 4 (0.5)	A: 5 (8.8)<br>B: 7 (12)<br>C: 63 (7.2)	A: 2 (3.5)<br>B: 3 (5.2)<br>C: 32 (3.7)	NR	NR	NR	NR

Author, year	Placental Abruption N (%)	GHTN N (%)	PE N (%)	GDM N (%)	SGA N (%)	PROM N (%)	Macrosomia
Negro et al. (2005)	NR	NR	NR	NR	NR	NR	NR

NR: Not reported, GDM: gestational diabetes mellitus, GHTN: gestational hypertension, PE: pre-eclampsia; PROM: pre-labour rupture of membranes, SGA = small-for-gestational-age, RPL: Recurrent Pregnancy Loss, SCH: subclinical hypothyroid, $TPOAb^+$: thyroid peroxidase antibody positive

Supplementary Table S5: Neonatal Outcomes in Studies Evaluating AITD and Pregnancy

Author, year	Neonatal admission N (%)	Gestational age Mean (SD)	Asphyxia neonatorum	Survival 28 days of neonatal life N (%)	Birth head circumference (cm) Mean (SD)	Birth weight (gm) Mean (SD)	Neonate height (cm) Mean (SD)	Neonatal thyroid function median (IQR)
Van Dijk et al. (2022)	NR	NR	NR	A: 47/69 (68.1)<br>B: 45/73 (61.6)	NR	NR	NR	NR
Riaz et al. (2022)	NR	NR	NR	NR	NR	NR	NR	NR
Leng et al. (2022)	NR	NR	<u>PRL group = 3 (0.5)</u><br>RPL+SCH:<br>LT4 = 0<br>Control = 2 (3.1)<br>RPL+TPOAb+:<br>LT4 = 0<br>Control = 0<br><br><u>Normal group = 10 (1.4)</u><br>Normal+SCH<br>LT4 = 0<br>Control = 1 (1.4)<br>Normal+TPOAb+<br>LT4 = 0	NR	NR	NR	NR	NR

Author, year	Neonatal admission N (%)	Gestational age Mean (SD)	Asphyxia neonatorum	Survival 28 days of neonatal life N (%)	Birth head circumference (cm) Mean (SD)	Birth weight (gm) Mean (SD)	Neonate height (cm) Mean (SD)	Neonatal thyroid function median (IQR)
			Control = 1 (2.9)					
Amiri et al. (2022)	147 (8.18)	GA at first week: 11.64 (4.18) GA at delivery: 39.01 (1.67)	NR	NR	34.75 (1.58)	3212.29 (454.92)	50.03 (2.24)	Neonate FT41 1st trimester: 2.9 (2.5-3.5) 2nd trimester: 3.3 (2.8-4.0) 3rd trimester: 2.8 (2.4-3.3)
Nazarpour (2017)	A: 2 (3.6) B: 12 (20.7) C: 75 (8.0)	A: 39.3 (1.3) B: 38.4 (1.7) C: 39.4 (1.4)	NR	NR	A: 34.5 (1.1) B: 34.9 (1.4) C: 34.7 (1.6)	A: 3139.1 (287.6) B: 3127.7 (523.5) C: 3236.6 (448.8)	A: 49.5 (1.7) B: 50.3 (1.5) C: 50.1 (2.3)	Neonatal TSH A: 1.3 (0.45-1.9) B: 1.0 (.43-1.9) C: 0.90 (0.40-1.7)
Kim et al. (2011)	NR	NR	NR	NR	NR	NR	NR	NR
Negro et al. (2006)	NR	NR	NR	NR	NR	NR	NR	NR
Negro et al. (2005)	NR	NR	NR	NR	NR	NR	NR	NR

Supplementary Table S6:

Question: [AITD] compared to [No AITD] for [Pregnant women]

Certainty assessment							№ of patients		Effect		Certainty	Importance
№ of studies	Study design	Risk of bias	Inconsistency	Indirectness	Imprecision	Other considerations	[AITD]	[No AITD]	Relative (95% CI)	Absolute (95% CI)		
**Preterm Delivery**												
3	randomised trials	not serious	not serious	serious[a]	not serious	none	33/156 (21.2%)	131/1924 (6.8%)	**OR 3.92** (2.54 to 6.05)	**155 more per 1,000** (from 88 more to 238 more)	⊕⊕⊕◯ Moderate[a]	
**Miscarriage**												
3	randomised trials	not serious	very serious[b]	serious[a]	serious[c]	none	13/156 (8.3%)	83/1924 (4.3%)	**OR 1.27** (0.16 to 9.82)	**11 more per 1,000** (from 36 fewer to 264 more)	⊕◯◯◯ Very low[a,b,c]	
**Placental Abruption**												
3	randomised trials	not serious	not serious	serious[a]	very serious[d]	none	1/156 (0.6%)	10/1924 (0.5%)	**OR 2.10** (0.45 to 9.84)	**6 more per 1,000** (from 3 fewer to 44 more)	⊕◯◯◯ Very low[a,d]	

CI: confidence interval; **OR:** odds ratio

Explanations

a. Populations varied (euthyroid TPOAb+, subclinical hypothyroidism, recurrent pregnancy loss, ART settings),
b. Conflicting directions across studies and high statistical heterogeneity ($I^2 = 89\%$)
c. Pooled CI wide and includes no effect.
d. Rare events, wide CI spanning substantial harm to no effect; effect estimate very imprecise.

Supplementary Table S7:

Question: [AITD Patients treated with LT4] compared to [Non-treated Patients] for [Pregnant women]

Certainty assessment							№ of patients		Effect		Certainty	Importance
№ of studies	Study design	Risk of bias	Inconsistency	Indirectness	Imprecision	Other considerations	[AITD Patients treated with LT4]	[Non-treated Patients]	Relative (95% CI)	Absolute (95% CI)		
**Miscarriage**												

Certainty assessment							№ of patients		Effect		Certainty	Importance
№ of studies	Study design	Risk of bias	Inconsistency	Indirectness	Imprecision	Other considerations	[AITD Patients treated with LT4]	[Non-treated Patients]	Relative (95% CI)	Absolute (95% CI)		
5	randomised trials	not serious	not serious	serious[a]	not serious	publication bias strongly suspected[b]	80/316 (25.3%)	110/322 (34.2%)	**OR 0.52** (0.34 to 0.80)	**129 fewer per 1,000** (from 192 fewer to 48 fewer)	⊕⊕◯◯ Low[a,b]	
**Preterm Delivery**												
4	randomised trials	not serious	not serious	serious[a]	not serious	publication bias strongly suspected[b]	14/223 (6.3%)	36/229 (15.7%)	**OR 0.37** (0.17 to 0.80)	**93 fewer per 1,000** (from 126 fewer to 27 fewer)	⊕⊕◯◯ Low[a,b]	
**Live birth**												
3	randomised trials	not serious	very serious[c]	serious[a]	serious[d]	publication bias strongly suspected[b]	118/129 (91.5%)	100/226 (44.2%)	**OR 10.28** (0.58 to 183.62)	**448 more per 1,000** (from 127 fewer to 551 more)	⊕◯◯◯ Very low[a,b,c,d]	

CI: confidence interval; **OR:** odds ratio

Explanations

a. Populations and interventions were heterogeneous (euthyroid TPOAb+, SCH, RPL, ART; varying TSH/antibody thresholds and timing of LT4), limiting applicability to a single, specific target population.
b. Few trials contribute to the outcome, positive trials cluster geographically, and one trial stopped early, increasing the plausibility of selective publication or exaggerated effects
c. large variability in individual trial point estimates and high heterogeneity (I^2 = 92%).
d. Pooled CI very wide, includes both substantial benefit and no effect.
